# Preosteoblast Adhesion and Viability Study of Freeze-Dried Bovine Bone Block Scaffold Coated with Human Umbilical Cord Mesenchymal Stem Cell Secretome

**DOI:** 10.1055/s-0044-1787105

**Published:** 2024-07-16

**Authors:** Arum Nur Kartika Putri, David Buntoro Kamadjaja, Andra Rizqiawan, Muhammad Subhan Amir, Ni Putu Mira Sumarta, Dewi Kartikawati Paramita

**Affiliations:** 1 Department of Clinical Medicine, Faculty of Medicine, Universitas Airlangga, Surabaya, Indonesia; 2 Department of Oral and Maxillofacial Surgery, Faculty of Dental Medicine, Universitas Airlangga, Surabaya, Indonesia; 3 Department of Histology and Cell Biology, Faculty of Medicine, Universitas Gadjah Mada, Yogyakarta, Indonesia

**Keywords:** bovine bone scaffold, secretome, bioactivity, adhesion, viability

## Abstract

**Objectives** Combining a three-dimensional scaffold with growth factors before implantation is one method used to increase scaffold bioactivity in bone tissue engineering. The mesenchymal stem cell (MSC)–conditioned medium (CM), called secretome, contains many proteins and growth factors required for tissue repair and growth. This study evaluated the bioactivity of a bovine bone scaffold combined with the secretome of human umbilical cord MSCs (hUC-MSCs) by analyzing MC3T3-E1 cell adhesion and viability on the scaffold.

**Materials and Methods** This *in vitro* laboratory study evaluated the effect of hUC-MSC secretome applied to bovine bone scaffolds processed using various techniques on MC3T3-E1 cell adhesion and viability. The three experimental groups included deproteinized bovine bone mineral–secretome (DBBM-CM), freeze-dried bovine bone–secretome (FDBB-CM), and decellularized FDBB-CM, whereas the control group was treated with DBBM alone. The cell adhesion test was performed using the centrifugation method after 6 and 24 hours, whereas the cell viability test was conducted using the trypan blue exclusion method after 24, 48, and 72 hours. Cell attachment was visualized after 4′,6-diamidino-2-phenylindole staining and viewed under inverted fluorescence microscopy.

**Stastical Analysis** Statistical analyses were performed using one-way analysis of variance, followed by a post hoc test in cases of significant differences.

**Results** Statistical analyses showed significantly greater adhesion of the preosteoblasts to the FDBB-CM scaffold at 6 hours ( *p* = 0.002). The results of the adhesion test at 24 hours and the viability tests at all observation times showed no significant differences ( *p* > 0.05). This study found that the average MC3T3-E1 cell adhesions and viabilities were highest for the FDBB-CM and DBBM-CM scaffolds. DBBM scaffolds with the secretome had better cell adhesion and viability than those without the secretome.

**Conclusion** The addition of MSC secretome increased bovine bone scaffold bioactivity especially in DBBM and FDBB scaffolds.

## Introduction

Bone grafting, the second most common tissue grafting procedure performed annually, 1 is necessary to treat defects caused by trauma, infection, or tumors. Bone defects can generally undergo regeneration; however, defects between tissues must support this process. This is called a critical-sized defect, the smallest intraosseous wound that cannot heal independently during its lifetime. In such cases, healing must be aided by a bone graft procedure that requires an ideal bone graft material. 2

Autologous bone graft is the gold standard because it has osteogenic, osteoinductive, and osteoconductive properties, but it has disadvantages such as postoperative donor-site morbidity and limited availability. The disadvantages of autologous bone grafts led to the development of bone tissue engineering (BTE). 3 Three main factors in BTE are required in bone regeneration: first, a scaffold to facilitate cell repopulation; second, growth factors to stimulate new tissue regeneration; and third, osteogenic cells to facilitate new bone matrix formation. 4

An alternative for BTE development is the use of xenograft scaffolds. Xenografts are used in countries where allografts are not permitted. One xenograft scaffold created of natural materials is the bovine bone xenograft. 5 Many types of bovine bone xenografts have been developed owing to their abundance and easy availability. Bovine bone-processing methods for xenograft scaffolds have been developed using various procedures. The heating and chemical extraction processes used to remove all organic components result in deproteinized bovine bone mineral (DBBM), the freeze-drying process results in freeze-dried bovine bone (FDBB), and the addition of the decellularization process results in decellularized FDBB (dcFDBB). 6 7 8 The scaffolds in BTE must be three-dimensional (3D) to support cell growth. These 3D scaffolds can provide a specific architecture, namely, a matrix that provides temporary mechanical support for cell migration, proliferation, and differentiation. 9

Scaffold materials have been widely developed in combination with mesenchymal stem cells (MSCs) to increase signaling factors in bone grafts and make them more similar to autografts. MSC cultures can be used as cell or cell-free therapies in the secretome (conditioned medium [CM]) and then combined in biomaterial scaffolds to support BTE efforts. 10 11 The use of the secretome has various advantages because it contains many growth factors and cytokines that can increase the tissue's angiogenic potential and anti-inflammatory effects to encourage bone regeneration. 12 The secretome can be obtained from various MSCs including human umbilical cord MSCs (hUC-MSCs), which has several advantages, namely, a customizable amount of nonliving secretome can be used directly in bone tissue defects, and it is more easily stored and transported before use. 13

The combination of scaffold components and the secretome of hUC-MSCs is a development in BTE; therefore, it must meet functional demands such as biocompatibility, biodegradability, appropriate porosity, favorable surface characteristics, ideal mechanical properties, and good bioactivity. This study aimed to evaluate the capacity of MSC secretome to increase the bioactivity of several types of bovine bone scaffolds in terms of cell migration, adhesion, viability, and proliferation.

## Materials and Methods

### Ethical Clearance and Preparation Material for Study

The ethics committee of the Faculty of Dental Medicine, Universitas Airlangga (632/HRECC.FODM/VIII/2022) approved the study protocol on August 25, 2022. The BATAN Research Tissue Bank produced scaffolds with dimensions of 9 × 9 × 9 mm (adhesion test) and 5 × 5 × 3 mm (viability test). The scaffolds made of bovine cancellous bone were cleaned with high-pressure water and immersed in methanol:chloroform at 1:1 ratio for 3 hours. The FDBB and dcFDBB scaffolds were prepared by soaking in 3% hydrogen peroxide for 3 hours, followed by rinsing with sterile distilled water. For the dcFDBB scaffold, hydrogen peroxide solution was mixed with the anionic surfactant sodium lauryl ether sulfate, followed by freeze drying for 15 hours. For the DBBM scaffold, the processed bovine bone was heated at 900°C for 3 hours. The process was continued with packaging and sterilization using 25 kGy gamma radiation. This study was conducted at the Stem Cell Development and Research Center, Institute of Tropical Disease (ITD), Universitas Airlangga, Indonesia.

### MC3T3-E1 Cell Culture Preparation

MC3T3-E1 cells were obtained from C57BL/6 mouse calvaria (ECACC 99072810; Sigma-Aldrich, St. Louis, MO, United States). The cells were frozen at −80°C and thawed gently in a 37°C water bath for 2 minutes. The cells were cultured in culture flasks with cell growth medium consisting of α modification of minimum essential medium eagle (α-MEM) (Sigma-Aldrich), 10% fetal bovine serum (Gibco BRL, Gaithersburg, MD, United States), and 2 mM L -glutamine. The cultures were incubated in 5% CO _2_ at 37°C. After 3 days of culture, the medium was changed and nonadherent cells were removed. After cell confluence reached 80%, trypsinization was performed using 0.05% ethylenediaminetetraacetic acid (Sigma-Aldrich) to obtain sufficient cells for research.

### Application of the Secretome on Scaffolds

hUC-MSC secretome was obtained from the finished preparations at the Stem Cell Development and Research Center, ITD, Universitas Airlangga, Indonesia. The treatment scaffolds for the adhesion and viability tests were placed in a sterile 24-well plate. The secretome was applied to the scaffold by the administration of 40 μL per side for the viability test scaffold and 370 μL per side for the adhesion test scaffold using a micropipette. The control scaffold involved the application of α-MEM medium at the same volumes. The applied scaffolds were incubated for 24 hours (37°C temperature, 98% humidity, and 5% CO _2_ ).

### MC3T3-E1 Cell Seeding on Scaffolds

An MC3T3-E1 cell suspension of 3 × 10 ^5^ cells/400 mL for the adhesion test scaffolds and 2 × 10 ^5^ cells/100 mL for the viability test scaffolds was prepared using the pipetting technique to create a homogeneous distribution. Scaffolds seeded with preosteoblasts were soaked in fresh medium. The cells were incubated for 6 and 24 hours for the adhesion test and for 24, 48, and 72 hours for the viability test (37°C temperature, 98% humidity, and 5% CO _2_ ). 5 14

### Cell Adhesion Evaluation

The adhesion tests were performed using centrifugation. The incubated scaffold was transferred into a 15-mL conical tube and a new medium was added to submerge the scaffold. The centrifugation was performed at 500 *g* for 5 minutes. The number of cells attached to the scaffold was counted by staining with 0.4% trypan blue (#1450021; Bio-Rad, California, United States) in a cell-counting chamber under a light microscope.

### Cell Viability Evaluation

Viability was tested using the trypan blue exclusion method. The scaffolds incubated for 24, 48, and 72 hours were trypsinized for 6 minutes. The number of live cells was counted using an automated cell counter (TC20TM Automated Cell Counter; Bio-Rad) after staining with 0.4% trypan blue ((#1450021; Bio-Radon dual-chamber cell-counting slides.

### 4′,6-Diamidino-2-phenylindole Staining

This test uses a scaffold measuring 5 × 5 × 3 mm. After secretome application, cell seeding, and incubation, the scaffold was transferred to a new well plate. The cells were fixed with 300 μL of 4% paraformaldehyde for 15 minutes. The liquid was discarded, and the cells were washed with phosphate buffered saline–Tween (Sigma-Aldrich). Next, 300 μL of 4′-6-diamino-2-phenylindole (DAPI; Thermo Scientific, Burlington, Canada) stain was added. The cells were incubated for 5 minutes, washed with phosphate buffered saline–Tween, and then observed under inverted fluorescence microscopy (CKX53; Olympus). The cell nuclei attached to the scaffold were stained blue using the DAPI stain.

### Statistical Analysis

Data were analyzed using SPSS software version 26 (IBM Corp., Armonk, New York, United States). The analysis began with a normality test using the Shapiro–Wilk's test, followed by Levene's test for homogeneity. One-way analysis of variance (ANOVA) was used to determine whether group differences existed. If a significant difference was identified, a post hoc test was used to determine which group was significant.

## Results

The MC3T3-E1 cell adhesion assay was performed by counting the number of cell adhesions on the scaffolds after centrifugation. The cells were counted using trypan blue staining under light microscopy. The normality test (Shapiro–Wilk's test) showed that all data were normally distributed ( *p* > 0.05), whereas the homogeneity test (Levene's test) showed that all data were homogeneous ( *p* > 0.05). The cell adhesion test showed significant differences between the study groups at 6 hours of observation (one-way ANOVA, *p* < 0.05), whereas no significant differences were noted at 24 hours of observation (one-way ANOVA, *p* > 0.05) ( Table 1 ). The difference test at 6 hours of observation was continued using a post hoc test. The post hoc results showed a significant difference in the average number of adherent MC3T3-E1 cells in the FDBB-CM versus dcFDBB-CM, DBBM-CM, and DBBM groups at 6 hours of observation ( *p* < 0.05) ( Table 2 ).

**Table 1:** Normality, homogeneity, and difference tests of cell adhesion

Observation time point (h)	Study group	Mean ± SD (×10 ^4^ cell/mL)	Normality ( *p* -value)	Homogeneity ( *p* -value)	One-way ANOVA ( *p* -value)
6	FDBB-CM	11.75 ± 2.36	0.220 a	0.426 a	0.002 b
	dcFDBB-CM	6.25 ± 0.96	0.272 a		
	DBBM-CM	6.50 ± 1.73	0.195 a		
	DBBM	6.00 ± 1.83	0.714 a		
24	FDBB-CM	7.25 ± 1.26	0,406 a	0.297 a	0.255
	dcFDBB-CM	6.00 ± 0.82	0.683 a		
	DBBM-CM	8.50 ± 2.38	0.051 a		
	DBBM	7.25 ± 1.71	0.850 a		

Abbreviations: ANOVA, analysis of variance; DBBM, deproteinized bovine bone mineral; DBBM-CM, deproteinized bovine bone mineral–secretome; dcFDBB-CM, decellularized freeze-dried bovine bone–secretome; FDBB-CM, freeze-dried bovine bone–secretome; SD, standard deviation.

a *p* > 0.05 (nonsignificant difference on normality and homogeneity tests).

b *p* < 0.05 (significant difference on ANOVA).

**Table 2:** Post hoc test results by study group at 6 hours of cell adhesion

Observation time (h)	Study group	Study group comparison	Significance ( *p* -value)
6	FDBB-CM	dcFDBB-CM	0.001 a
		DBBM-CM	0.001 a
		DBBM	0.001 a

Abbreviations: DBBM, deproteinized bovine bone mineral; DBBM-CM, deproteinized bovine bone mineral–secretome; dcFDBB-CM, decellularized freeze-dried bovine bone–secretome; FDBB-CM, freeze-dried bovine bone–secretome.

a *p* < 0.05 (significant difference).

The trypan blue exclusion test to determine MC3T3-E1 cell viability on the scaffold showed the highest average cell viability in the FDBB-CM group at 6 hours and the DBBM-CM group at 24 and 72 hours. The normality test (Shapiro–Wilk's test) showed that all data were normally distributed ( *p* > 0.05), and the homogeneity test (Levene's test) showed that all data were homogeneous ( *p* > 0.05). One-way ANOVA showed that cell viability did not differ significantly between groups at any observation time point ( *p* > 0.05) ( Table 3 ).

**Table 3:** Normality, homogeneity, and difference tests of cell viability

Observation time (h)	Study group	Mean ± SD (×10 ^4^ cell/mL)	Normality ( *p* )	Homogeneity ( *p* -value)	One-way ANOVA ( *p* -value)
24	FDBB-CM	4.94 ± 2.39	0.219 a	0.164 a	0.277
	dcFDBB-CM	3.29 ± 0.55	1.000 a		
	DBBM-CM	4.75 ± 2.22	0.728 a		
	DBBM	2.20 ± 1.45	0.365 a		
48	FDBB-CM	5.83 ± 1.14	0.466 a	0.072 a	0.434
	dcFDBB-CM	3.86 ± 2.36	0.223 a		
	DBBM-CM	6.03 ± 0.55	0.990 a		
	DBBM	5.12 ± 2.07	0.249 a		
72	FDBB-CM	6.58 ± 1.10	0.995 a	0.529 a	0.436
	dcFDBB-CM	4.94 ± 1.10	1.000 a		
	DBBM-CM	6.95 ± 1.38	0.784 a		
	DBBM	6.40 ± 2.22	0.728 a		

Abbreviations: ANOVA, analysis of variance; DBBM, deproteinized bovine bone mineral; DBBM-CM, deproteinized bovine bone mineral–secretome; dcFDBB-CM, decellularized freeze-dried bovine bone–secretome; FDBB-CM, freeze-dried bovine bone–secretome; SD, standard deviation.

a *p* > 0.05 (nonsignificant difference on normality and homogeneity tests).

**Fig. 1:**
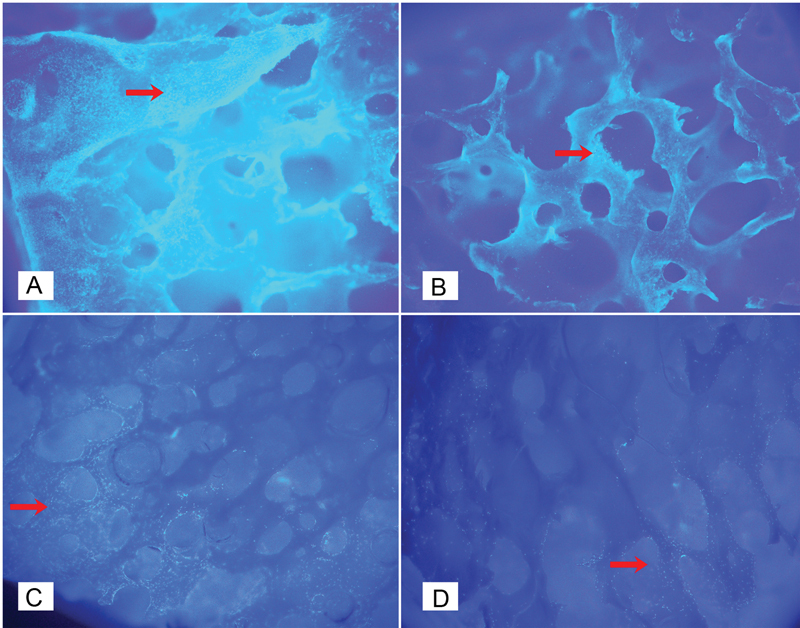
DAPI staining results at 24 hours. DAPI staining was observed using an inverted fluorescence microscope (CKX53; Olympus) at ×40 magnification. Red arrows indicate cell adhesion on the scaffold surface at 24 hours. (A) Freeze-dried bovine bone–secretome, (B) decellularized bovine bone–secretome, (C) deproteinized bovine bone mineral–secretome, and (D) deproteinized bovine bone mineral alone. DAPI, 4′,6-diamidino-2-phenylindole.

All scaffolds showed different surface morphologies based on observations using a fluorescence microscope at ×40 magnification. At the 24-hour observation, cell attachment to the scaffold occurred. The cell nuclei of MC3T3-E1 cells were stained by DAPI fluorescence staining and emitted blue fluorescence under inverted fluorescence microscopy ( Fig. 1 ).

**Fig. 2:**
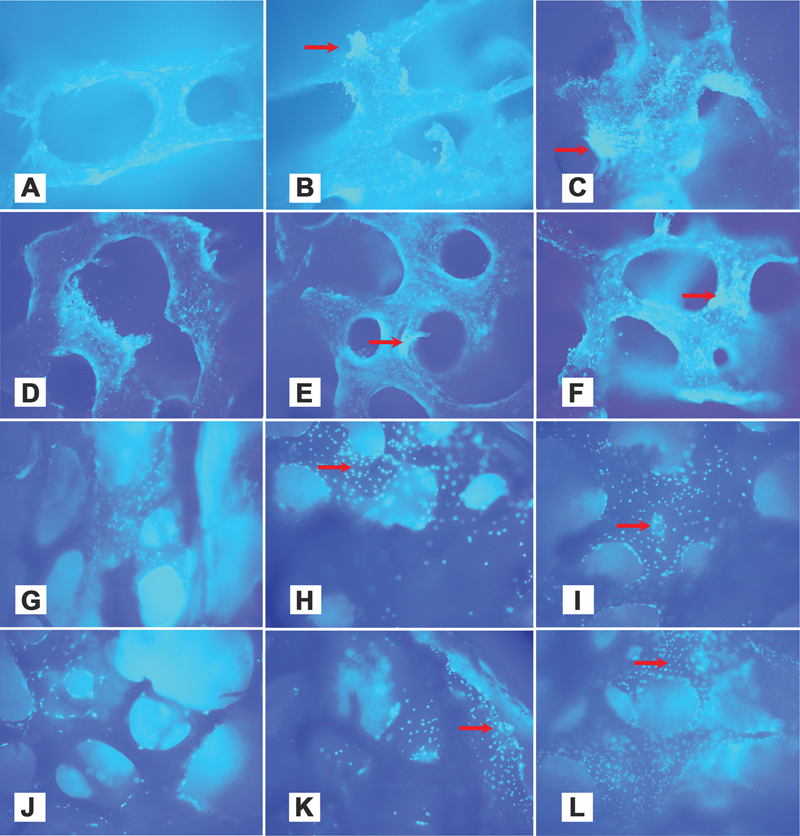
4′,6-Diamidino-2-phenylindole staining results at 24, 48, and 72 hours. Cell adhesion increased at each observation time point for all study groups (left to right: 24, 48, and 72 hours). Red arrows indicate cell colonies. (A–C) Freeze-dried bovine bone–secretome, (D–F) decellularized bovine bone–secretome, (G–I) deproteinized bovine bone mineral–secretome, and (J–L) deproteinized bovine bone mineral alone.

The DAPI fluorescence observation results supported the cell adhesion test results, namely, the degree of cell adhesion was the same for all study groups, with the highest cell colonization in the FDBB-CM versus the dcFDBB-CM, DBBM-CM, and DBBM groups at each observation time point ( Fig. 2 ).

**Fig. 3:**
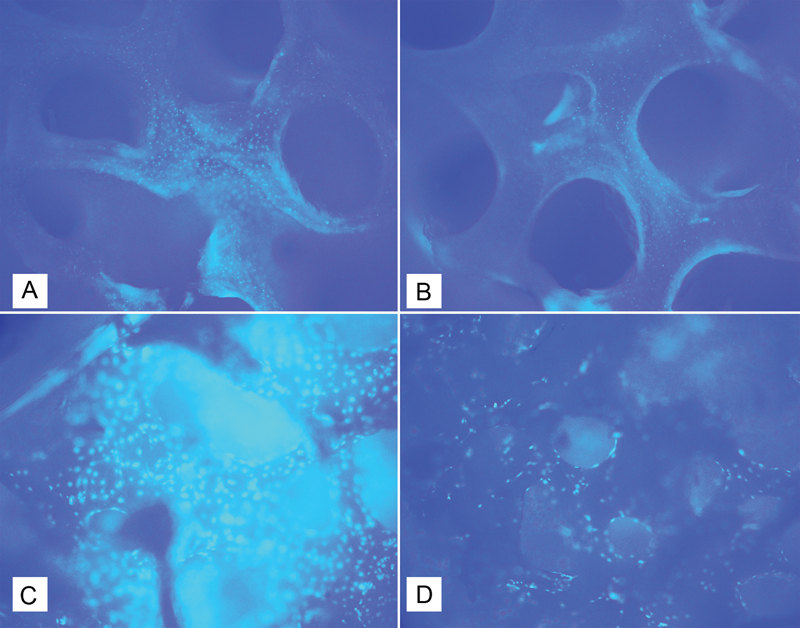
4′,6-Diamidino-2-phenylindole staining results at 72 hours. The adhesion of MC3T3-E1 cells to the top surface of the scaffold at 72 hours. (A) Freeze-dried bovine bone–secretome, (B) decellularized bovine bone–secretome, (C) deproteinized bovine bone mineral–secretome, and (D) deproteinized bovine bone mineral alone.

Cell attachment occurred on the top surface of the scaffold in all research groups. Most MC3T3-E1 cells attached to the top surface of the scaffold in the DBBM scaffold regardless of secretome addition, whereas fewest attached to the dcFDBB-CM scaffold ( Fig. 3 ).

## Discussion

The main objective of this study was to evaluate scaffold bioactivity in BTE by testing cell adhesion and viability on bovine bone scaffolds with secretome addition. The scaffolds used in this study consisted of 3D xenografts of bovine cancellous bone created using freeze-dried, deproteinated, and decellularized processes. The 3D scaffold functions as a matrix or analog of the extracellular matrix (ECM), which acts as a physical support structure and regulator of biological activities such as cell adhesion, migration, proliferation, and differentiation. 9 15 Xenografts must undergo processes to preserve ECM structure, composition, function, and bioavailability for improving clinical success. 16

DBBM, a bovine bone xenograft, is a scaffold used for comparison with the FDBB and dcFDBB scaffold groups in this study. 17 The process of creating DBBM scaffolds involves heating to remove organic components and reduce its immunogenic potential to ensure good biocompatibility. The osteoinduction ability of DBBM is lost, but its osteoconduction properties persist because of the porosity and interconnection of the hydroxyapatite crystalline structure. 18 19

Preparation of the FDBB and dcFDBB scaffolds through freeze-drying aims to remove the cell components of the tissue. The bone material is subjected to a rapid freezing cycle and extensive lyophilization by dehydration via sublimation. Intracellularly formed ice crystals during freezing cause cell death and disrupt surface antigens, reducing the risk of immunogenic reactions. 3 20 FDBB has osteoinductive and osteodifferentiation potential because it retains the organic components, growth factors, glycosaminoglycans, noncollagenous ECM proteins, and scaffold morphology. 8 21

Cell adhesion is a complex dynamic process involving the adsorption of proteins onto surfaces and expression of specific peptide sequences. Cells attach to certain surfaces via integrins and usually die if they fail to attach. The ECM contains proteins that are recognized by integrins and cell receptors such as arginine–glycine–aspartic acid ligand, fibrinogen, collagen, vitamin C protein, fibronectin, and vitronectin. These ligands regulate cell physiological processes triggered by the ECM, including migration, adhesion, growth, and apoptosis. 22 23

Scaffolds with included secretome tend to have a higher average number of cell adhesions, possibly due to the contents of secretome molecules such as galectin 9, vascular cell adhesion molecule-1, intercellular adhesion molecule-1, and intercellular adhesion molecule-4, which support the adhesion process. Galectin 9 exhibits various biological functions, such as triggering cell aggregation, which supports cell adhesion and proliferation. Vascular cell adhesion molecules and intercellular adhesion molecules affect cell functions, regulating cell growth and adhesion between cell molecules. This secretory effect is also associated with increased fibronectin levels in the tissues involved in cell adhesion. 23 24 25 Growth factors produced by the hUC-MSC secretome also support cell migration, adhesion, survival, proliferation, and differentiation. 26

The freeze-drying and decellularization processes leave organic components, such as ECM, on the scaffold. Various molecules in the ECM play a role in the mechanisms supporting the cell adhesion process. 22 Fibronectin, one such ECM component, is an ECM protein that promotes cell adhesion to the FDBB scaffolds. The binding of fibronectin and other adhesion proteins to cell surface receptors increases cell spreading, focal contact formation, and adhesion strength. At 6 hours after the implantation of cells onto a scaffold containing fibronectin, the presence of actin filaments, which support the adhesion process, was observed. 27

The dcFDBB-CM scaffold exhibited the least cell adhesion. The washing stage used an anionic surfactant solution on the dcFDBB scaffold to remove DNA components from the tissue by destroying protein bonds and lysing cell membranes, thus damaging the scaffold's organic components. The destruction of organic material eventually damages the ECM, affecting its components that support the adhesion process. Therefore, the osteoinduction properties of FDBB scaffolds are superior to those of dcFDBB scaffolds because they are supported by suitable organic components. 8

However, the observations at 24 hours differed. The DBBM-CM scaffold showed the highest average number of adherent cells, but the differences were not statistically significant. This is because the fibronectin contained in FDBB does not affect cell adhesion at 12 hours after cell implantation. 27 The concentration of fibronectin on the surface can decrease continuously owing to the competitive adsorption–desorption process of serum proteins. 28

Cell growth material and surface micropore morphology are other essential factors that influence cell adhesion. Pore size is the main factor influencing cell adhesion. 22 The porosity of the DBBM scaffold exceeds those of the FDBB and dcFDBB scaffolds; however, the differences are not statistically significant. The FDBB, dcFDBB, and DBBM scaffolds have mean pore sizes of 412 ± 12, 450 ± 31, and 511 ± 58 μm, respectively. 29 The minimum pore size for significant bone growth is 75 to 100 μm, and many studies have suggested the need for pores to be >300 μm to enable bone formation and vascularization. All scaffolds used in this study had optimal pore sizes for new bone formation. The scaffolds with larger pore sizes showed the highest percentage of cell adhesion because they had higher infiltration rates and even cell distributions. The cells migrated from the scaffold's edge to its center, indicating that cell migration increased with increasing pore size. 30 Average pore size, size distribution, pore volume, interconnectivity, and pore shape are essential parameters in scaffold design. The scaffold must be highly porous to allow cell growth and support neovascularization. 31

The viabilities of the MC3T3-E1 cells implanted in the scaffolds in this study did not differ significantly after 24, 48, or 72 hours. The MC3T3-E1 cells adhered to all scaffolds and formed colonies on their surfaces, indicating their viability ( Fig. 2 ). Cell–matrix interactions are critical to the regulation of cell structure, growth, and differentiation. Cell survival or viability depends on interactions with the ECM, other cells, and growth factors in the growth medium. If this interaction is prevented, cells can undergo apoptosis as a physiological form of programmed cell death. 32 The addition of a growth factor-rich hUC-MSC secretome also supports the role of the adhesion process in cell survival. The secretome causes increased levels of Akt, vascular endothelial growth factor, and transforming growth factor-β, which led to increased cell proliferation and migration. Therefore, the application of a growth factor-containing secretome is believed to support cell survival. 23

The FDBB scaffolds contained organic components that supported cell adhesion and viability. Adhesive proteins such as fibronectin and laminin-1 are the most effective at increasing cell survival. 32 Therefore, the good cell viability noted on the FDBB-CM scaffolds may be due to the presence of organic components such as fibronectin and collagen and the addition of a secretome containing growth factors such as hepatocyte growth factor, transforming growth factor-β, vascular endothelial growth factor, catalase, heme oxygenase-1, B cell lymphoma-2, Akt, and hypoxia-inducible factor-1. 23 A good adhesion process supports cell viability on the scaffold. The low cell viability on dcFDBB-CM scaffolds was also caused by the low cell adhesion owing to destruction of the organic materials. 8

The high cell viability on the DBBM-CM scaffold was supported by direct cell adhesion to the scaffold. The direct adhesion process is supported by the surface topography characteristics, such as pore size and degree of roughness. The DBBM scaffold has the largest pore size, which results in the highest percentage of cell adhesion and superior cell infiltration and migration to those of the other scaffolds. 29 33 The highest degree of cell adhesion facilitates greater subsequent cell proliferation and migration. The significantly greater number of cells on the scaffold with the highest porosity leads to higher cell proliferation and migration on the scaffold, leading to greater cell viability. 30 MC3T3-E1 cell infiltration and migration to the middle and top of the DBBM-CM scaffold occurred more quickly in the current study than in other studies ( Fig. 3 ). 34

The results of this study showed that the FDBB and DBBM scaffolds had better bioactivity than the dcFDBB scaffold as evidenced by their superior cell adhesion and viability. The addition of the secretome in this study increased scaffold bioactivity. The study limitations include the composition of the secretome used and the degree of scaffold surface roughness.

## Conclusion

Despite the limitations of this study, we conclude that coating the bovine bone block scaffolds with MSC secretome increased bioactivity as evidenced by improved cell adhesion, viability, and proliferation, with the FDBB scaffold being comparable to the DBBM scaffold. Further research must measure the growth factor levels of the secretome and evaluate the *in vivo* scaffold bioactivity of all available bovine bone scaffolds.
